# Acute Appendicitis Due to Enterobius vermicularis Infestation: A Case Report

**DOI:** 10.7759/cureus.76535

**Published:** 2024-12-28

**Authors:** Ravi Chandra Tata, Mahmoud Abdelreheem, Aina Mercant Osuna, Sudhakar Mangam

**Affiliations:** 1 Department of General Surgery, East Kent Hospitals University NHS Foundation Trust, Queen Elizabeth The Queen Mother Hospital, Margate, GBR

**Keywords:** antihelminthic, enterobius vermicularis infestation, histology post appendicectomy, laparoscopic appendicectomy, parasitic appendicitis

## Abstract

Acute appendicitis is the most frequent abdominal surgical emergency worldwide. While luminal obstruction due to fecaliths and lymphoid hyperplasia is a common cause, parasitic infections are a rare but significant contributor. *Enterobius vermicularis*, the most common helminthic infection in developed countries, can trigger appendiceal inflammation through a mechanical obstruction or immune response.

This report presents a case of a 16-year-old male with acute appendicitis secondary to *E. vermicularis* infestation. The patient presented with typical appendicitis symptoms, and laparoscopic appendectomy revealed the parasitic involvement, confirmed by histopathology. Postoperative recovery was uneventful following surgical intervention and anti-helminthic treatment.

*E. vermicularis* is often an incidental finding in appendectomy specimens but may play a pathogenic role in some cases of appendicitis. This case underscores the importance of considering parasitic infections in patients with atypical presentations of appendicitis and highlights the value of histopathological examination in appendectomy cases.

## Introduction

Acute appendicitis is the most common cause of abdominal surgical emergencies worldwide, with an annual incidence of approximately 100 per 100,000 individuals [1]. The condition typically results from luminal obstruction caused by fecaliths, lymphoid hyperplasia, or, less commonly, neoplasms or parasitic infections. Risk factors for acute appendicitis include younger age, dietary habits, and genetic predispositions [2]. Left untreated, appendicitis can progress to perforation, peritonitis, or abscess formation, emphasizing the importance of timely diagnosis and surgical intervention [3].

*Enterobius vermicularis*, commonly known as pinworm, is the most frequently encountered helminthic infection in developed countries, including the United Kingdom. While often asymptomatic, this parasite can rarely cause appendicitis through luminal obstruction or by triggering localized inflammation [4]. This case report describes an instance of acute appendicitis secondary to *E. vermicularis* infestation in a 16-year-old male, underscoring the importance of recognizing parasitic involvement in atypical presentations of appendicitis.

This article was previously presented as a poster abstract at the Seventh East Kent Surgical Conference on November 27, 2024.

## Case presentation

A 16-year-old male presented to the emergency department with a two-day history of worsening right lower quadrant abdominal pain, rated 9 out of 10 on a pain scale, accompanied by nausea and anorexia. He denied any bowel or urinary symptoms and had no significant past medical history, recent abdominal trauma, or travel history. His social history revealed that he is a football player and lives with his parents and three younger siblings, all under the age of 10.

On physical examination, localized and rebound tenderness were noted in the right iliac fossa (RIF). Initial laboratory tests revealed no elevated inflammatory markers, and a urine dip showed traces of blood and leukocytes.

Given the persistent pain and urine dip findings, the management options of watchful waiting, diagnostic laparoscopy, or computed tomography of the kidneys, ureter, and bladder (CT KUB) to rule out renal pathology were discussed with the patient. He opted for the CT KUB, which failed to positively identify the appendix and showed no evidence of renal or ureteric pathology.

**Figure 1:**
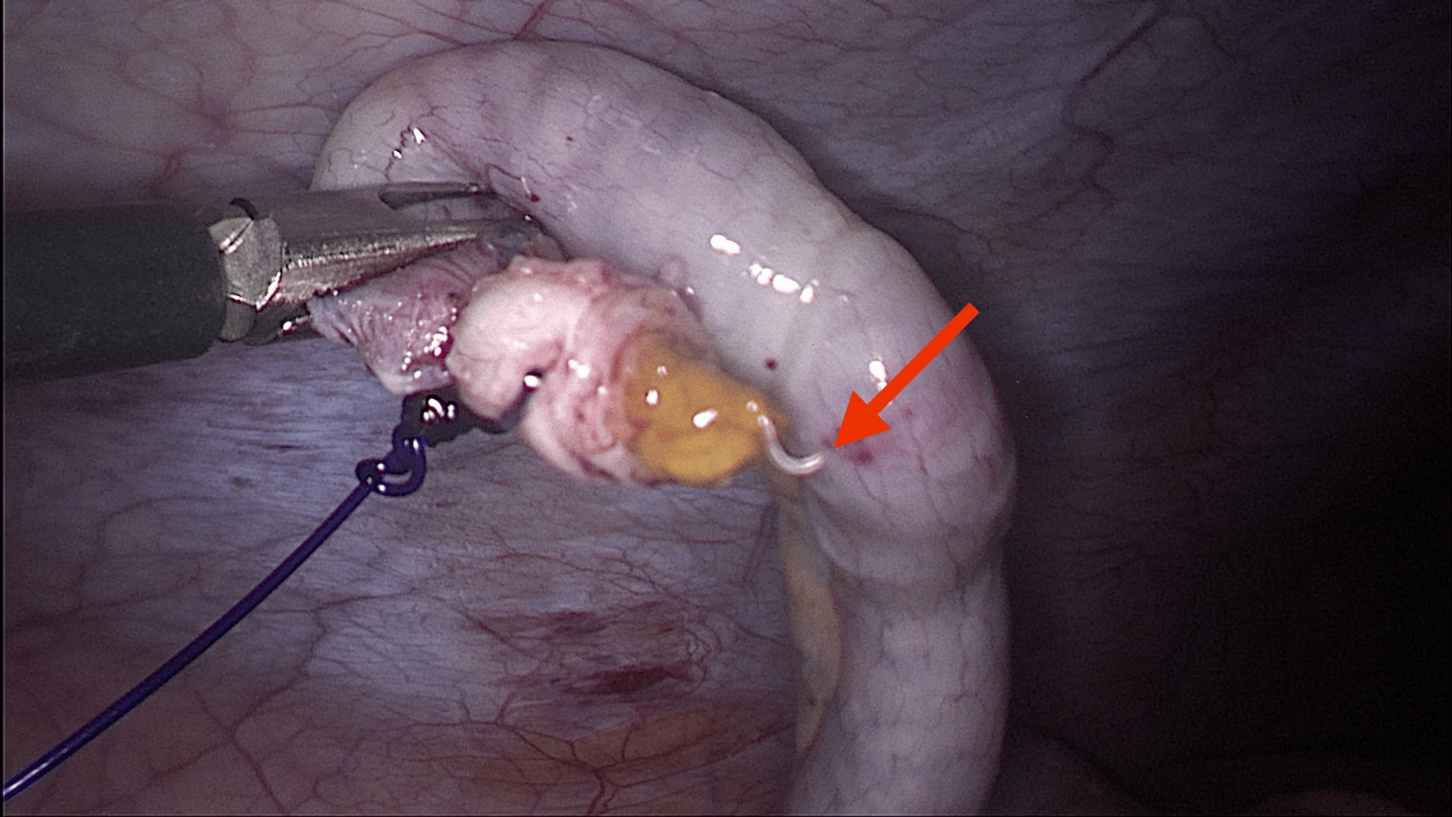
Intraoperative image of live parasitic worm (red arrow) in the appendiceal lumen.

As the pain persisted and repeat blood tests revealed a mildly elevated C-reactive protein (CRP) of 23 mg/L, surgical intervention was deemed necessary. The patient underwent a diagnostic laparoscopy and appendectomy. Intraoperatively, the appendix appeared grossly normal with no obvious signs of inflammation; however, several live parasitic worms were discovered within the appendiceal lumen (Figure 1) and stump. Prompt suctioning of the stump was performed to prevent peritoneal soiling with the live worms, and the appendix was retrieved in an endobag.

Postoperative recovery was uneventful, and the patient was discharged on postoperative day one. Anti-helminthic therapy with mebendazole was initiated upon discharge after discussion with a microbiologist. The patient's general practitioner was notified to provide prescriptions for the rest of the patient's family.

## Discussion

Enterobius vermicularis

*E. vermicularis*, commonly referred to as pinworm, is a parasitic infection predominantly affecting children aged 5 to 14 years. According to the CDC, approximately 40 million cases occur annually in the United States, while in the UK, parasitic threadworm infestations are estimated to affect 20% to 30% of children in preschool and primary school age, although exact prevalence rates remain uncertain [1,2].

Life cycle and transmission

Transmission occurs through the ingestion of eggs via the fecal-oral route, with humans serving as the definitive host. Once ingested, the eggs hatch in the small intestine, and larvae migrate to the ileum and cecum, where they mature into adults over a two-month period [3]. Adult worms reside in the ileum and cecum, with females migrating nocturnally to the perianal area to deposit eggs. This migration is responsible for the characteristic pruritic symptoms associated with infection [4]. Scratching due to pruritus contaminates the fingers, leading to autoinfection through ingestion of eggs. Eggs can also contaminate surfaces, facilitating infection among household members [4]. Rarely, retro-infection occurs when larvae hatch near the perianal region and migrate back into the gastrointestinal tract, completing the life cycle [5].

Clinical manifestations

Infection with *E. vermicularis* is often asymptomatic; however, perianal pruritus is a common symptom, particularly at night. In rare cases, *E. vermicularis* has been implicated in conditions such as vulvovaginitis, pelvic and peritoneal granulomas, and eosinophilic colitis. Despite these associations, its role in appendicitis remains controversial [6].

Relationship to appendicitis

The potential mechanisms by which *E. vermicularis* may contribute to appendicitis include mechanical obstruction of the appendiceal lumen, triggering appendiceal colic, or direct mucosal invasion causing inflammation [7]. Histopathological changes associated with *E. vermicularis-*related appendicitis vary widely, ranging from normal appendices to severe ulceration and perforation [8].

The prevalence of *E. vermicularis* in appendicitis has been well-documented, with a meta-analysis estimating an overall prevalence of 4.2% in appendectomy cases [5]. A literature review reported that *E. vermicularis-*associated appendicitis accounts for approximately 1% of cases in children [6]. Of these, 27% had grossly normal appendices, whereas 68.7% demonstrated histological evidence of inflammation. This variability underlines the importance of histological examination in suspected cases [9].

Relevance to the present case

This case adds to the growing body of literature on acute appendicitis caused by *E. vermicularis* but offers unique aspects that set it apart. While parasitic infestation of the appendix has been documented, the diagnostic challenges presented in this patient highlight the variability in clinical presentations and the importance of a high index of suspicion for atypical causes of appendicitis.

The patient’s clinical presentation, including a two-day history of RIF pain rated at 9/10 and associated with anorexia and nausea, was typical for appendicitis. The patient had no bowel or urinary symptoms. However, due to the urine dip's positive findings for blood and leukocytes, initial concerns arose regarding urinary tract pathology. Ultrasound showed no abnormalities, and CT imaging failed to visualize the appendix. This underscores how non-specific symptoms and equivocal imaging findings can delay diagnosis in cases of parasitic appendicitis.

The clinical course of this patient, with only mildly raised inflammatory markers (CRP 11 on admission, rising to 23) and negative imaging, raises questions about the threshold for intervention. Cases like this challenge clinicians to balance the risks of watchful waiting with early surgical intervention, particularly in patients with non-specific imaging findings.

In this case, the patient was asymptomatic for pruritus despite having significant *E. vermicularis* infestation. The appendix appeared grossly normal during surgery, but microscopic inflammation was evident upon histological examination. This finding reinforces the hypothesis that *E. vermicularis* may incite subclinical inflammation, potentially contributing to appendicitis without overt clinical or gross pathological signs [6].

**Figure 2:**
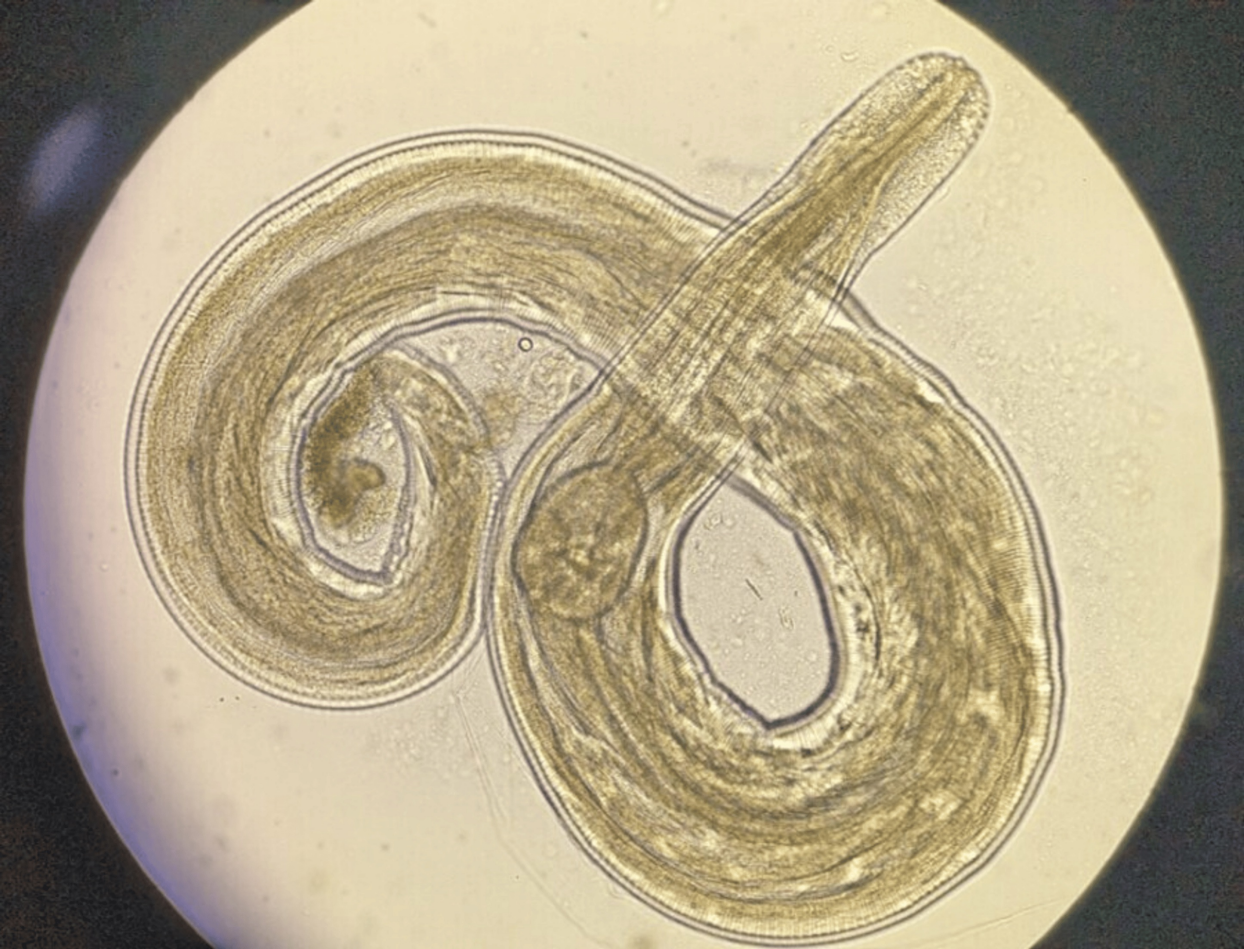
Microscopic image of Enterobius vermicularis identified within the appendiceal tissue.

The histopathological findings in this case suggest *E. vermicularis* as the potential causative agent, with acute inflammation and direct observation of the parasite within the appendiceal lumen (Figure 2). This aligns with prior studies that suggest mechanical obstruction and local immune responses mediated by *E. vermicularis* as mechanisms of appendiceal inflammation [6,7]. However, this case's diagnostic complexity is a reminder that parasitic appendicitis may not always fit the classic clinical or imaging patterns.

*E. vermicularis* is not typically endemic in industrialized countries, yet its prevalence remains significant due to its ease of transmission, particularly in group settings like schools and sports teams. Although the patient denied recent travel, his participation in football, a contact sport, and his residence with younger siblings, a population known to be susceptible to parasitic worm infections, raise the possibility of environmental exposure. Pinworm infections are often underdiagnosed due to their nonspecific symptoms and the stigma associated with parasitic infections, highlighting the need for a thorough social and activity history in evaluating patients with appendicitis [10].

This case provides a unique opportunity to examine the practical and multidisciplinary considerations in managing acute appendicitis caused by *E. vermicularis*. While parasitic appendicitis is rare, the intraoperative finding of live worms emerging from the appendix added a layer of complexity. Prompt suctioning was employed to minimize peritoneal contamination, which is a critical step in preventing post-operative complications such as abscess formation or diffuse peritonitis. Although such scenarios are uncommon, they underscore the need for surgeons to be prepared to manage unexpected findings intraoperatively.

Current literature supports strategies to minimize peritoneal contamination in these cases. Techniques such as endostapling or employing a triple-end loop method to seal the appendix prior to transection have been suggested as ways to contain luminal contents effectively, including worms [11]. While immediate suctioning was employed in this case, preoperative knowledge of the parasitic etiology through diagnostic stool tests or imaging might have allowed for tailored intraoperative techniques, further reducing contamination risk. This emphasizes the importance of considering parasitic causes in patients, especially children and adolescents, with atypical presentations of appendicitis.

Another notable aspect of this case was the immediate involvement of a microbiologist postoperatively. Before histopathological confirmation, the team initiated discussions with a microbiologist to determine anti-helminthic treatment for the patient and their household contacts. This approach is crucial because pinworm infections often involve asymptomatic carriers within close contact, and untreated household members may act as reservoirs, leading to reinfection. Initiating treatment without waiting for biopsy results expedited patient and family management and reduced the risk of recurrence [12].

The necessity of a multidisciplinary approach in such cases cannot be overstated. Early involvement of microbiologists ensures that patients receive appropriate anti-parasitic therapy tailored to the organism and the clinical scenario. Additionally, educating families about hygiene practices, such as frequent handwashing and laundering of bed linens, is vital in preventing reinfection. This approach aligns with guidelines advocating a holistic strategy to address both the immediate surgical condition and the underlying parasitic infection [10].

## Conclusions

In conclusion, this case underscores the diagnostic challenges and variable clinical presentations of acute appendicitis caused by *E. vermicularis*. It highlights the importance of considering parasitic infections in patients with atypical findings or intraoperative evidence of parasitic involvement. A comprehensive approach integrating clinical, radiological, and pathological data is essential for accurate diagnosis and effective management. Furthermore, it emphasizes the importance of obtaining a detailed history, particularly focusing on risk factors and symptoms associated with parasitic worm infections, especially in children and younger adults, who are more susceptible to such infestations.

This case emphasizes the value of prompt surgical intervention, meticulous intraoperative techniques to minimize contamination, and early multidisciplinary input, including anti-helminthic therapy and public health measures. By addressing both the clinical and social aspects of parasitic infections, this case serves as a teaching tool for surgeons and underscores the importance of a thorough histopathological examination in appendectomy specimens to guide tailored patient care.
